# Interpretable machine learning models for predicting perioperative myocardial injury in non-cardiac surgery

**DOI:** 10.1093/ehjdh/ztag093

**Published:** 2026-06-12

**Authors:** Benjamin Sailer, Sibel Sari-Yavuz, Stephanie Biergans, Raphael Verbücheln, Lars-Christian Achauer, Peter Rosenberger, Michaela Hardt, Michael Koeppen

**Affiliations:** Medical Data Integration Center, University Hospital Tübingen, Tübingen, Germany; Department of Anesthesiology and Intensive Care Medicine, University Hospital Tübingen, Hoppe-Seyler-Straße 3, Tübingen 72076, Germany; Medical Data Integration Center, University Hospital Tübingen, Tübingen, Germany; Medical Data Integration Center, University Hospital Tübingen, Tübingen, Germany; Medical Data Integration Center, University Hospital Tübingen, Tübingen, Germany; Department of Anesthesiology and Intensive Care Medicine, University Hospital Tübingen, Hoppe-Seyler-Straße 3, Tübingen 72076, Germany; Medical Data Integration Center, University Hospital Tübingen, Tübingen, Germany; Department of Anesthesiology and Intensive Care Medicine, University Hospital Tübingen, Hoppe-Seyler-Straße 3, Tübingen 72076, Germany

**Keywords:** Perioperative myocardial injury, Non-cardiac surgery, Machine learning, Explainable boosting machines, Risk prediction model

## Abstract

**Aims:**

Perioperative myocardial injury (PMI) is a frequent and often asymptomatic complication after non-cardiac surgery and is associated with increased short- and long-term mortality. Conventional risk scores, such as the Revised Cardiac Risk Index (RCRI), have limited predictive accuracy and are infrequently used in clinical practice. We aimed to develop and temporally validate an interpretable machine learning model using Explainable Boosting Machines (EBMs) to predict PMI from routine pre-operative data.

**Methods and results:**

In this retrospective cohort study at a tertiary care centre in Germany, we included 9323 adult patients undergoing 9824 non-cardiac surgical procedures between 2014 and 2023 who received post-operative high-sensitivity cardiac troponin testing as part of routine care. PMI was defined as a post-operative elevation of high-sensitivity cardiac troponin above the upper reference limit. An EBM was trained on structured pre-operative data from 2014 to 2021 and evaluated in a temporally independent test cohort from 2022 to 2023, with performance compared with logistic regression, random forest, XGBoost, and a modified RCRI. Model discrimination, calibration, and Brier scores were assessed. Feature contributions were examined using internal shape functions and SHAP values. PMI occurred in 2804 procedures (28.5%). The EBM achieved the highest predictive performance (AUROC 0.730, 95% CI 0.720–0.740), outperforming all comparators. Calibration was robust across clinically relevant risk ranges. Key predictors included age, leukocyte count, renal function, potassium, and platelet count. The EBM identified high-risk patients more efficiently than the modified RCRI and ESC guideline-based strategies (Number Needed to Evaluate 3.0 vs. 3.5) and reduced troponin assays by 18.2% in the temporally independent cohort.

**Conclusion:**

An interpretable machine learning model trained on routine clinical data can accurately predict PMI and outperform existing risk scores. The EBM supports individualized risk stratification and may enhance perioperative decision-making and resource allocation within a guideline-directed testing population. Prospective and external validation is required before clinical implementation.

## Graphical Abstract

**Graphical Abstract:**
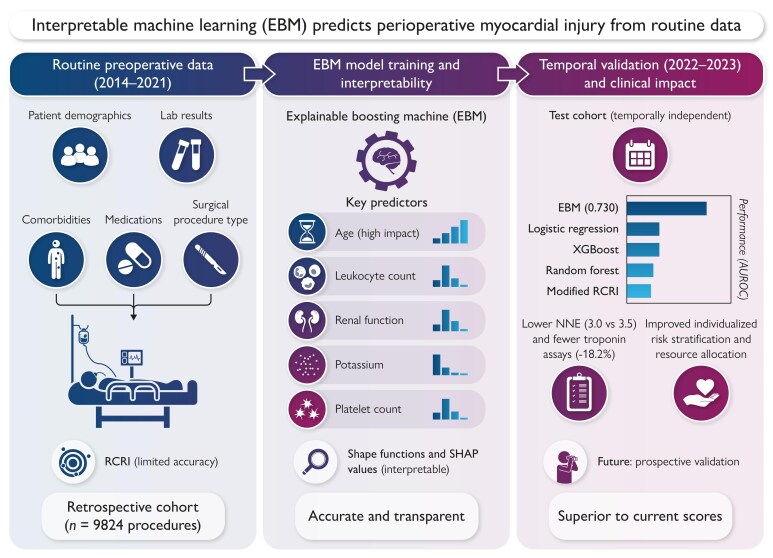

## Introduction

Perioperative complications remain a leading cause of morbidity and mortality worldwide, despite major advances in surgical safety and perioperative care. Among these, perioperative myocardial injury (PMI) has emerged as a major contributor to adverse outcomes after non-cardiac surgery. PMI is common, frequently asymptomatic, and associated with a markedly increased risk of both short-term and long-term mortality. In large prospective studies, the incidence of PMI ranges from 8% to 16% depending on the population and detection strategy, with 30-day mortality exceeding 9% in affected patients.^1,2^ As surgical volumes increase and populations age, the burden of PMI is expected to rise further.

To address this challenge, accurate identification of patients at elevated risk is essential. Several cardiac risk indices have been developed to support perioperative risk stratification, including the Revised Cardiac Risk Index (RCRI)^3^ and the Myocardial Infarction or Cardiac Arrest (MICA)^4^ calculator derived from the National Surgical Quality Improvement programme. However, the performance of these tools is limited, particularly in unselected or high-risk surgical populations. Studies have shown that the RCRI underestimates risk in vascular surgery patients and lacks adequate discrimination and calibration across diverse cohorts.^5,6^ Moreover, traditional risk scores often rely on a limited set of pre-defined variables and yield only coarse, categorical risk classes rather than continuous, individualized estimates. Combined with the need for manual data entry, this reduces their precision and clinical utility, contributing to low adoption in routine practice.^7–9^

Machine learning (ML) offers new opportunities to improve perioperative risk assessment by analyzing large volumes of structured healthcare data and uncovering complex relationships among variables. Gradient boosting models and other ensemble methods have demonstrated high predictive accuracy in retrospective studies of post-operative complications, including PMI.^10,11^ Nevertheless, most of these models lack interpretability, making it difficult for clinicians to understand or trust individual predictions. In clinical settings where decisions carry significant consequences, transparent and explainable models are essential for adoption and regulatory acceptance.^12^

Explainable Boosting Machines (EBMs) are a class of inherently interpretable machine learning models that combine the flexibility of non-linear modelling with the transparency of additive structures. They build upon generalized additive models by combining gradient boosting of shallow decision trees with bagging techniques, and can visualize the individual effect of each predictor for clinical interpretation.^13^ This enables clinicians to assess model behaviour, identify meaningful risk factors, and integrate model outputs into clinical decision-making. EBMs are well suited for healthcare applications where both accuracy and interpretability are required.^14,15^

In this study, we developed and evaluated an interpretable machine learning model based on EBMs to predict perioperative myocardial injury after non-cardiac surgery. Using a large and clinically heterogeneous cohort from a tertiary care hospital, we trained the model exclusively on structured data routinely available in electronic health records. We compared its performance to established clinical risk scores and widely used machine learning algorithms, and assessed its discrimination, calibration, and clinical utility. Our aim was to demonstrate that interpretable machine learning can support individualized perioperative risk stratification and provide a scalable foundation for clinical deployment.

## Methods

### Study population and ethical considerations

This retrospective study was conducted at the University Hospital of Tübingen and included 9323 adult patients who underwent 9824 non-cardiac surgical procedures between 2014 and 2023. Eligibility required at least one post-operative high-sensitivity cardiac troponin (hs-cTn) measurement to enable identification of perioperative myocardial injury (PMI). Demographic data, and pre-operative laboratory values were extracted from electronic health records, while diagnostic and procedural information was extracted from administrative billing data. The study was approved by the local ethics committee (IRB# 287/2023BO2), with a waiver of informed consent in accordance with institutional policy on pseudonymized data use.

### Inclusion criteria and data extraction

Patients were included if they underwent non-cardiac surgery during the study period and had a post-operative hs-cTn measurement available within the routine clinical workflow. Patients under 18 years, with STEMI diagnosis, or without post-operative troponin data were excluded. Consequently, model development reflects patients selected for post-operative troponin testing within routine clinical care and therefore estimates PMI risk conditional on this guideline-directed testing strategy. PMI was defined as any post-operative hs-cTn concentration above the upper reference limit (LOINC 10839-9: 0.04 μg/L, LOINC 89579-7: 40 ng/L), regardless of clinical symptoms. Data extraction covered age, sex, relevant ICD-10 and OPS codes, and laboratory parameters obtained within seven days before surgery. All predictor variables were strictly limited to data available before the index surgical procedure. No intraoperative, post-operative, or outcome-related variables were included in model training. The resulting structured dataset included surgical metadata, diagnostic codes, and routine pre-operative laboratory values.

### Data pre-processing

We conducted the data pre-processing steps prior to model training. For this, we categorized laboratory data into two pre-operative intervals: 0–2 days and 3–7 days prior to surgery. For each interval, we aggregated multiple laboratory measurements using the mean, minimum, maximum, standard deviation, and the difference between the last and first values. We aggregated pre-surgical ICD-10 diagnoses and OPS procedure codes, defined as occurring between 0 and 90 days prior to the index surgery; diagnoses recorded after surgery were excluded from model development. We merged surgical procedures occurring within a three-day window into a single observation. To prevent outcome leakage, we calculated a modified Charlson Comorbidity Index excluding the categories for acute myocardial infarction and congestive heart failure, as these conditions may overlap with perioperative myocardial injury definitions.

Values below the 2.5th percentile and above the 97.5th percentile were winsorized to these percentile limits. This approach was chosen to retain clinically relevant extreme values while reducing the impact of outliers that could adversely affect model training and stability of machine learning algorithms.^16–18^ Laboratory variables with more than 80% missingness were removed. We imputed missing data using a K-nearest neighbours or iterative imputer for logistic regression models and assigned a constant value of –999 for all other models. This encoding allows the absence of information to be treated as an informative category and ensures that all algorithms, including those without native support for missing values, can process the full dataset consistently. For logistic regression, we applied range normalization to scale numerical features to a 0–1 interval. Boolean variables were encoded as binary integers (0/1).

Modified revised cardiac risk index calculation and Charlson Comorbidity Index. A modified revised cardiac risk index (mRCRI) was calculated using the ICD codes as described by Andersson *et al*.,^19^ except for the pre-operative insulin use component, which was omitted due to data availability. The RCRI subsystems and their corresponding codes were defined as follows: ischaemic heart disease (ICD-10 I20–I25), history of cerebrovascular disease (ICD-10 I60–I69), history of congestive heart failure (ICD-10 I11.0, I42.0, I42.1, I50), elevated creatinine (defined as a diagnosis of renal disease or requirement for dialysis; ICD-10 N03, N04, N17–N19, R34, I12, I13, or Z99.2), and high-risk surgery (thoracic, intra-abdominal, or suprainguinal aortic procedures), specified as thoracic surgery (OPS 5-32–37), intra-abdominal surgery (OPS 5-43–54), and suprainguinal aortic surgery (OPS 5–38) (see Supplementary material online, *Tables S1* and *S3*).

Furthermore, a modified Charlson Comorbidity Index was calculated based on the algorithm from Sundararajan *et al*.^20^ the ICD-10 codes provided by Quan *et al*.^21^ and, with the subsystems for acute myocardial infarction and congestive heart failure omitted to prevent data leakage into the predictive models (see Supplementary material online, *Table S2*).

In accordance with clinical guidelines, troponin measurement is mandatory for surgeries classified as intermediate or high cardiac risk, as well as for low-risk procedures in patients aged 65 years or older or those with relevant cardiovascular comorbidities. Due to the absence of standardized ICD-10 codes for cardiac risk stratification, overall perioperative mortality risk was used as a proxy for cardiac risk. Surgical procedures and their associated mortality risks were evaluated using publicly available billing data from the German Institute for the Hospital Remuneration System (InEK) for the years 2019–2023. Surgeries were consolidated at higher hierarchical levels, and mortality risk was calculated for each procedure. Procedures were then categorized as high risk (>5% mortality rate), intermediate risk (1–5%), or low risk (<1%).

### Machine learning process

To predict perioperative myocardial injury, we trained an Explainable Boosting Machine (EBM) and compared its performance with three established machine learning algorithms: eXtreme Gradient Boosting (XGBoost),^22^ Random Forest (RF),^23^ and regularized logistic regression. In addition, we also calculated a modified version of the Revised Cardiac Risk Index (mRCRI), adapted to our dataset. All models were trained using data from patients included between 2014 and 2021 and evaluated on a held-out temporal test set comprising patients from 2022 to 2023. Outcome labels (PMI) were assigned exclusively based on post-operative troponin measurements and were not used as input features at any stage of model training.

Within the training period (2014–2021), stratified five-fold cross-validation was employed for hyperparameter tuning using a random search with 50 iterations (10 iterations for EBM due to slower training). After identifying the optimal hyperparameters, a single final model was trained and saved. This process was repeated five times with different random seeds, resulting in five models per architecture. Performance was assessed using area under the receiver operating characteristic curve (ROC-AUC), F1-score, balanced accuracy, precision, recall and specificity. Models were optimized based on the ROC-AUC, and decision thresholds were subsequently adjusted. Recalibration and class imbalance methods were assessed but deemed unnecessary.

The EBM is based on a generalized additive model (GAM) framework of the form $g(E[y])\;=\sum f_{i}(x_{i})\;+\;\sum f_{ij}(x_{i},\;\;x_{j})$, where g(⋅) is a function relating the output to predictors, each $f_{i}$ represents a univariate shape function corresponding to an individual predictor variable, and $f_{ij}$ refers to interaction functions modelling the joint effect of predictor pairs $x_{i}$ and $x_{j}$. This additive structure allows non-linear effects of each predictor to be visualized and interpreted independently, enhancing model transparency. EBM further extend this framework by incorporating pairwise interaction terms where appropriate. To improve robustness and accuracy, EBMs use gradient boosting applied to each feature, combined with bagging, where multiple simple models are trained on random subsets of the data.^24,25^

To enhance interpretability, variable importance and marginal effects were examined using EBM’s internal shape functions and validated with SHAP value analysis for the other models.^22^ Model discrimination was visualized using ROC curves with bootstrapped 95% confidence intervals (*n* = 1000). The mRCRI served as a clinical reference standard and was evaluated using the same procedure.

Data extraction and transformation from the FHIR server were performed in R 4.1.1 (R Core Team, 2024) using fhircrackr^26^ 2.2.0 and tidyverse 1.3.1.^25^ All modelling was performed in Python 3.8.10 (Python Software Foundation) using pandas 2.2.3,^27,28^ imblearn 0.11.0,^29^ scikit-learn 1.3.1^30^ (for RF, XGBoost, logistic regression, and CV), and the interpretML package 0.4.4 (for EBM).^13^ SHAP analyses were conducted with shap 0.43.0. Visualizations were generated in R 4.4.2 (R Core Team, 2024) using ggpubr 0.6.0,^31^ tidyverse 2.0.0,^25^ and patchwork 1.2.0^32^ and Python using matplotlib 3.10.1.^33^

The code used for this analysis will be made available as open source following publication.

### Generation of synthetic patients

To generate mock patients exclusively for illustration purposes (not for training or evaluating models) for each group (PMI and non-PMI), real patient records were randomly sampled from the respective cohorts. For each sampled patient, numerical features were perturbed by multiplying it with a random value between 0.95 and 1.05. Boolean and categorical variables were stochastically reassigned by sampling values from the empirical distribution of the respective feature within the same group (PMI or non-PMI).

## Results

### Study cohort and baseline characteristics

Of 296 928 surgical inpatient encounters during the study period, 9824 non-cardiac procedures underwent post-operative troponin testing and 287 104 encounters did not. Patients selected for post-operative troponin measurement were older with a mean age of 62.8 vs. 56.8 years, had a higher comorbidity burden with a modified Charlson Comorbidity Index of 1.10 vs. 0.82, and more frequently underwent high-risk surgery at 50.0% vs. 24.3% compared with surgical inpatients without troponin testing as shown in *Table 1*. These differences demonstrate enrichment of higher-risk individuals through guideline-directed testing.

**Table 1:** Baseline characteristics of patients with and without perioperative myocardial injury

Variable	Surgical inpatient encounters (*n* = 287 104)	Total cohort (*n* = 9824)	With PMI (*n* = 2804)	Without PMI (*n* = 7020)	*P*-value
**Demographics**					
Age (years; mean ± SD)	56.83 ± 18.89	62.75 ± 17.99	67.39 ± 16.33	60.90 ± 18.29	<0.001
Female sex (%)	161 581 (56.3%)	4534 (46.2%)	1180 (42.1%)	3354 (47.8%)	<0.001
**Comorbidities**					
Atrial fibrillation (%)	4850 (1.7%)	363 (3.7%)	135 (4.8%)	228 (3.2%)	<0.001
Chronic kidney disease (%)	4498 (1.6%)	274 (2.8%)	120 (4.3%)	154 (2.2%)	<0.001
Coronary artery disease (%)	5530 (1.9%)	408 (4.2%)	167 (6.0%)	241 (3.4%)	<0.001
Diabetes mellitus (%)	10 040 (3.5%)	478 (4.9%)	159 (5.7%)	319 (4.5%)	0.022
History of heart failure (%)	2183 (0.8%)	164 (1.7%)	68 (2.4%)	96 (1.4%)	<0.001
Hypertension (%)	23 454 (8.2%)	1082 (11.0%)	369 (13.2%)	713 (10.2%)	<0.001
Peripheral artery disease (%)	2324 (0.8%)	110 (1.1%)	41 (1.5%)	69 (1.0%)	0.053
Previous stroke/TIA (%)	831 (0.3%)	25 (0.3%)	5 (0.2%)	20 (0.3%)	0.468
**Laboratory Values (Pre-operative)**					
BNP or NT-proBNP^a^ (ng/L; mean + SD)	3606.39 ± 12505.98	5463.33 ± 15661.66	9342.05 ± 21258.89	2908.72 ± 9684.16	<0.001
Creatinine (mg/dL; mean + SD)	0.97 ± 1.16	1.07 ± 1.04	1.33 ± 1.33	0.96 ± 0.88	<0.001
CRP (mg/dL; mean + SD)	2.78 ± 5.22	5.03 ± 7.30	5.53 ± 7.81	4.84 ± 7.07	0.022
eGFR (mL/min/1.73 m2; mean + SD)	79.05 ± 18.85	74.76 ± 21.45	66.72 ± 25.08	77.94 ± 18.92	<0.001
Haemoglobin (g/dL; mean + SD)	12.68 ± 2.18	12.18 ± 2.51	11.55 ± 2.51	12.42 ± 2.46	<0.001
Haemoglobin POCT (g/dL; mean + SD)	11.87 ± 2.75	11.27 ± 2.41	10.99 ± 2.34	11.44 ± 2.44	<0.001
Troponin T (µg/L; mean + SD)	0.21 ± 1.69	0.20 ± 0.93	0.55 ± 1.60	0.04 ± 0.08	<0.001
high-sensitive Troponin T (ng/L; mean + SD)	108.16 ± 1086.01	161.63 ± 1218.57	628.64 ± 2401.28	9.71 ± 13.42	<0.001
**Perioperative Characteristics**					
Anaemia (Hb <10 g/dL; mean + SD)	18587 (6.5%)	1828 (18.6%)	727 (25.9%)	1101 (15.7%)	<0.001
High-risk surgery (%)	69,639 (24.3%)	4911 (50.0%)	1693 (60.4%)	3218 (45.8%)	<0.001
mod. Charlson Comorbidity Index (mean + SD)	0.82 ± 1.66	1.10 ± 1.94	1.28 ± 2.10	1.03 ± 1.87	<0.001
**Outcome**					
In-hospital Mortality (%)	3047 (1.1%)	766 (7.8%)	459 (16.4%)	307 (4.4%)	<0.001
Major Adverse Cardiovascular Events (%)	5875 (2.0%)	492 (5.0%)	244 (8.7%)	248 (3.5%)	<0.001

^a^Patients may be represented in more than one category if they underwent multiple procedures.

Within the tested cohort, perioperative myocardial injury occurred in 2804 procedures corresponding to 28.5%. Patients with perioperative myocardial injury were older with a mean age of 67.4 vs. 60.9 years and were more often male. Cardiovascular comorbidities including atrial fibrillation, chronic kidney disease, coronary artery disease, heart failure, and hypertension were more frequent in patients with perioperative myocardial injury with all *P* values below 0.001, whereas diabetes mellitus, peripheral artery disease, and prior stroke or transient ischaemic attack did not differ significantly.

Pre-operative laboratory values indicated higher cardiovascular risk in patients with PMI, reflected by elevated BNP or NT-proBNP, creatinine, and troponin levels, and lower estimated glomerular filtration rate (eGFR) and haemoglobin. High-risk surgery was more common in the PMI group (60.4% vs. 50%), as was a greater comorbidity burden (Charlson comorbidity index 1.28 vs. 1.10). Anaemia was present in 25.9% of PMI patients compared with 18.6% without PMI. PMI was associated with higher in-hospital mortality (16.4% vs. 7.8%; *P* < 0.001) and a significantly increased incidence of major adverse cardiovascular events (MACE, 8.7% vs. 5%; *P* < 0.001) (*Table 1*).

### Discriminative performance of machine learning models compared with clinical risk scores

Model performance was evaluated exclusively in the temporally independent test cohort comprising procedures performed between 2022 and 2023. The RCRI is commonly used during pre-operative anaesthesia assessments as a clinical screening tool for perioperative cardiovascular risk. We benchmarked its performance against multiple machine learning models across five random seeds.

**Figure 1:**
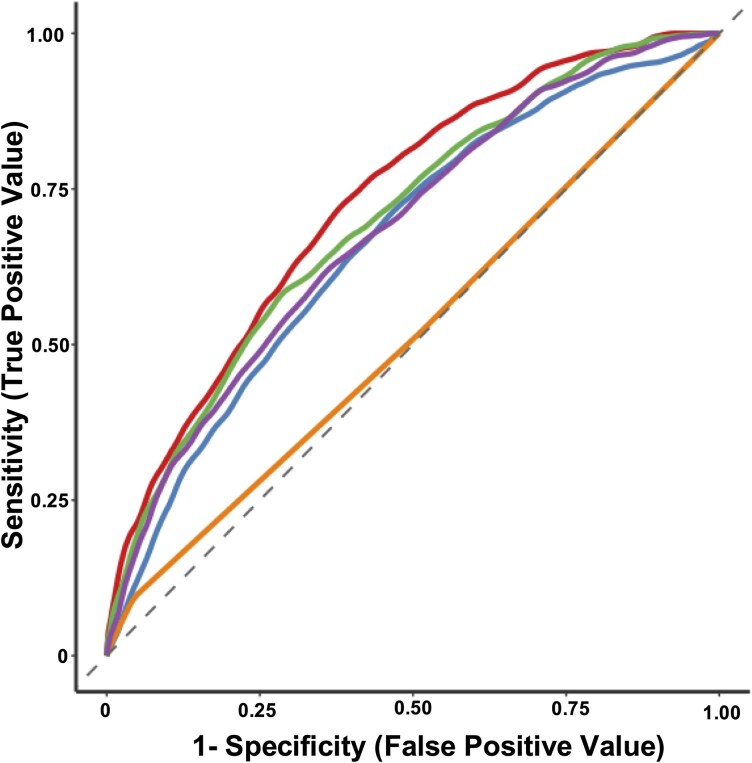
Discriminative performance of machine learning models and clinical scores for predicting perioperative myocardial injury. Bootstrapped receiver operating characteristic (ROC) curves comparing the Explainable Boosting Machine, XGBoost, Random Forest, logistic regression, and the modified Revised Cardiac Risk Index in the temporally independent validation cohort (2022–2023). The EBM achieved the highest area under the ROC curve (AUC), with machine learning models consistently outperforming the mRCRI across the full range of false-positive rates.

The EBM showed the highest predictive performance for perioperative myocardial injury, with an area under the receiver operating characteristic curve (AUCROC) of 0.730 (95% CI 0.720–0.740), outperforming logistic regression (0.665, 95% CI 0.655–0.676), Random Forest (0.703, 95% CI 0.693–0.713) and XGBoost (0.698, 95% CI 0.688–0.709). In contrast, the modified RCRI (mRCRI) showed significantly lower predictive accuracy (AUCROC 0.524, 95% CI 0.518–0.530) (*Table 2*, Supplementary material online, *Table S5*). ROC curves are presented in *Figure 1*, with bootstrapped confidence intervals for model performance in Supplementary material online, *Figure S2*.

**Table 2:** Performance of machine learning models for predicting perioperative myocardial injury

Model	AUROC (95% CI)	Balanced accuracy	F1 score	Precision	Recall	Specificity	Brier Score	Average Precision
**Explainable boosting machine**	0.730 (0.720–0.740)	0.662 ± 0.002	0.514 ± 0.003	0.432 ± 0.004	0.635 ± 0.018	0.69 ± 0.015	0.173 ± 0.0	0.373 ± 0.001
**Logistic regression**	0.665 (0.655–0.676)	0.622 ± 0.003	0.473 ± 0.004	0.373 ± 0.003	0.65 ± 0.024	0.594 ± 0.02	0.204 ± 0.002	0.337 ± 0.001
**XGBoost**	0.698 (0.688–0.709)	0.629 ± 0.008	0.465 ± 0.014	0.428 ± 0.01	0.51 ± 0.034	0.747 ± 0.022	0.196 ± 0.008	0.351 ± 0.006
**Random forest**	0.703 (0.693–0.713)	0.639 ± 0.007	0.483 ± 0.007	0.423 ± 0.015	0.565 ± 0.017	0.713 ± 0.024	0.181 ± 0.003	0.356 ± 0.007
**Mod. Revised Cardiac Risk Index**	0.524 (0.518–0.530)	0.524 ± 0.0	0.154 ± 0.0	0.436 ± 0.0	0.093 ± 0.0	0.955 ± 0.0	—	0.288 ± 0.0

All machine learning models achieved good discrimination, with the EBM consistently ranking highest across validation folds. Logistic regression demonstrated slightly inferior performance compared with the ensemble methods, likely attributable to the high dimensionality of the dataset. The EBM further provided model interpretability by visualizing individual feature effects and uncovering non-linear risk patterns.

### Model fairness

Given historic biases in the delay of diagnoses of women with cardiac diseases such as myocardial infarction, we assessed the model’s fairness for females. While females made up 46% of the cohort, they only contributed 42% of the PMI cases, see *Table 1*. Whether this is a result of a truly lower incidence or a bias in under detection as documented for post-operative myocardial infarction, needs to be determined in a prospective study. In terms of model performance we found minor differences identified between groups (see Supplementary material online, *Table S6*).

### Model calibration

**Figure 2:**
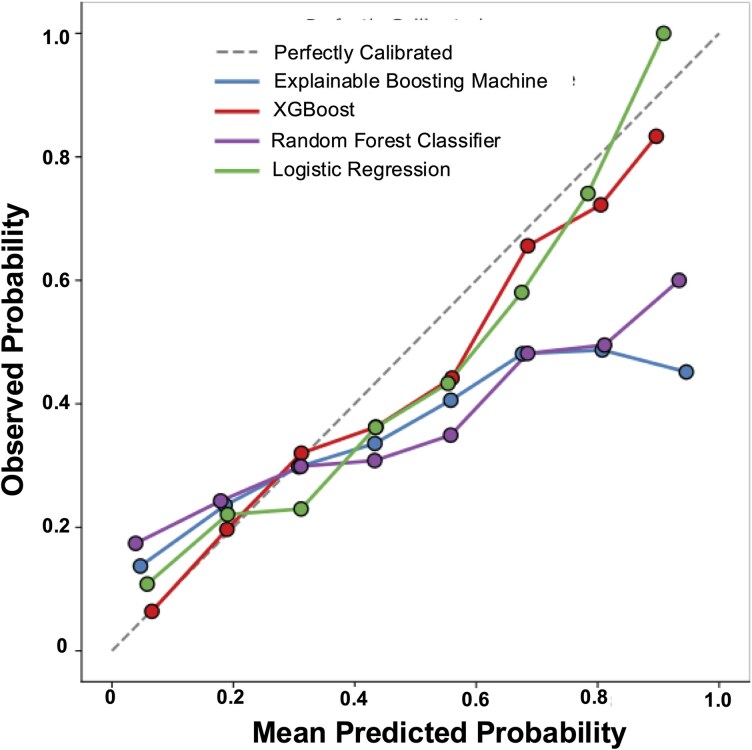
Calibration of machine learning models for predicting perioperative myocardial injury. Calibration plots comparing observed and predicted event rates across deciles of predicted risk in the temporally independent validation cohort (2022–2023). The Explainable Boosting Machine, XGBoost, Random Forest, and logistic regression models demonstrated good calibration across most risk ranges. Slight deviations at the highest risk deciles reflect the limited number of patients with very high predicted risk. Overall, the machine learning models showed consistent alignment between predicted probabilities and observed outcomes.

Calibration analysis revealed differences in the agreement between predicted and observed perioperative myocardial injury across models (*Figure 2*). The EBM (red line) and Random Forest (green line) demonstrated the closest overall calibration to the 45° reference line, particularly within the low-to-intermediate predicted probability range.

All models showed good calibration in the low-to-intermediate predicted risk ranges. In the upper deciles, calibration curves became more variable, with both EBM and Random Forest demonstrating clear superiority. Logistic regression and XGBoost maintained generally acceptable calibration across the range, but fell below it at medium-to-high risks, indicating a tendency to overpredict risk in those regions. Greater fluctuations in the medium-to-high risk bins for these models likely reflect data sparsity. Quantitative assessment using the Brier score supported these visual findings. The EBM achieved the lowest Brier score (0.173), followed by Random Forest (0.181), XGBoost (0.196), and logistic regression (0.204).

These findings underscore differences in calibration behaviour across model types. The EBM provided the most consistent probabilistic estimates across the clinically relevant risk spectrum, combining strong discriminative performance with robust calibration. In contrast, other methods such as Logistic Regression and XGBoost required more careful post-hoc calibration and exhibited greater variability, particularly at the upper end of the risk range.

### Feature importance and risk profiles

**Figure 3:**
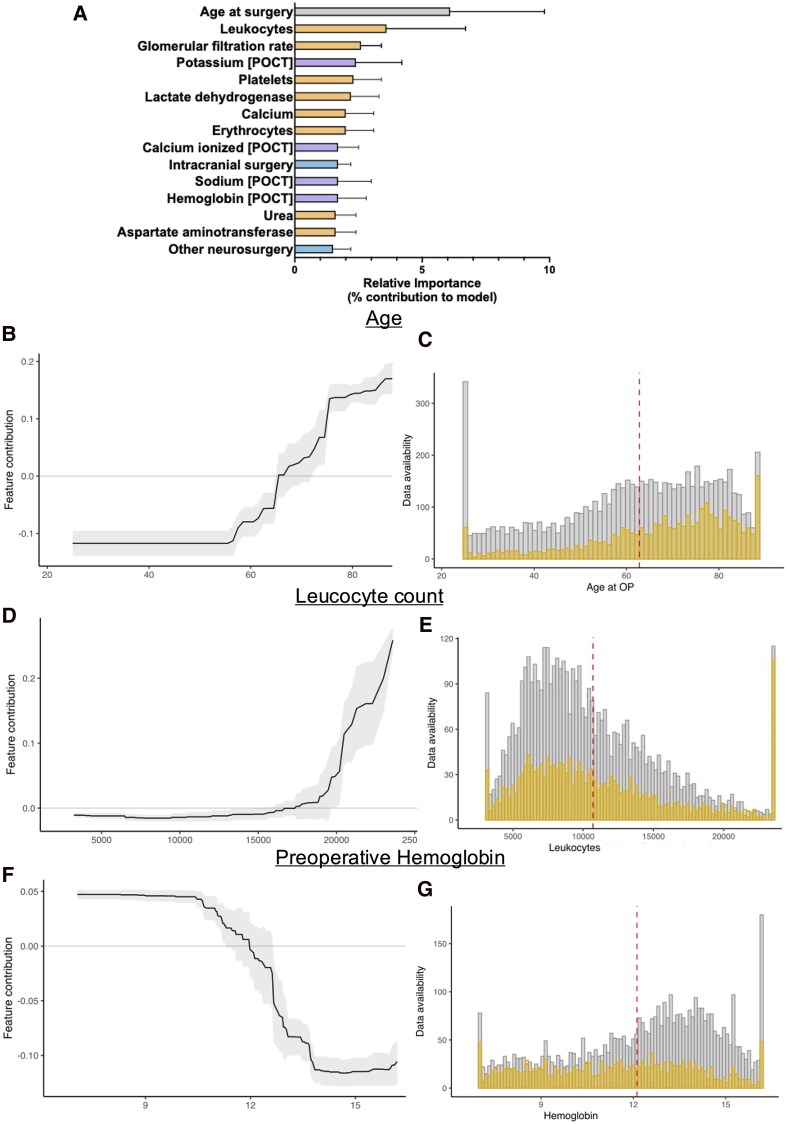
Feature importance and effect visualization for perioperative myocardial injury prediction. (*A*) Relative importance of the top predictors in the Explainable Boosting Machine model, expressed as percentage contribution to the model. Colours indicate laboratory values (orange), diagnoses (blue), point-of-care diagnostics (purple) and patient characteristics (grey). (*B*, *D*, *F*) Shape functions showing the marginal effect of age (*B*), leucocyte count (*D*), and pre-operative haemoglobin (*F*) on the log-odds of predicted perioperative myocardial injury risk. Shaded areas represent 95% confidence intervals. (*C*, *E*, *G*) Corresponding distributions of these features in patients with (yellow) and without (grey) perioperative myocardial injury. Non-linear risk patterns were observed for age and leukocyte count, while haemoglobin demonstrated a marked inverse non-linear association with increased risk at lower concentrations.

Feature importance analysis (*Table 3*) reports the average relative contributions across all 20 trained models (the EBM using its native importance metric and three comparator models, logistic regression, Random Forest and XGBoost, via SHAP) while *Figure 3A* displays only the EBM’s own feature importance. Age at surgery and leukocytes emerged as the strongest predictors in the multi model average, followed by glomerular filtration rate, potassium and platelets. Routine laboratory values such as lactate dehydrogenase, calcium, erythrocytes, calcium ionized and craniotomy with excision or destruction exhibited smaller but still meaningful contributions, with lower ranking features including sodium, haemoglobin, urea, aspartate aminotransferase or other operations on the skull, brain, and meninges. Individual feature effect plots demonstrated non-linear risk relationships. PMI risk increased markedly in older patients (particularly beyond age 75; *Figure 3B*) and rose sharply with leukocyte concentrations above clinical thresholds (*Figure 3D*). The relationship between haemoglobin and predicted PMI risk was non-linear, with a sharp increase in the lower reference values range, consistent with clinically relevant anaemia thresholds (*Figure 3F*). A smaller secondary increase was observed at very high haemoglobin concentrations, though this may be influenced by data sparsity in that range. The corresponding availability histogram (*Figure 3G*) confirms broad measurement of haemoglobin across the cohort, with sufficient density in both low and normal ranges to support robust inference.

**Table 3:** Top 15 predictors of perioperative myocardial injury ranked by mean importance across four machine learning models

Feature	Average importance
Age at surgery	0.061 ± 0.037
Leukocytes (26464-8)	0.036 ± 0.031
Glomerular filtration rate (62238-1)—2day (m…	0.026 ± 0.008
Potassium [POCT] (2823-3)	0.024 ± 0.018
Platelets (26515-7)	0.023 ± 0.011
Lactate dehydrogenase (14804-9)	0.022 ± 0.011
Calcium (2000-8)	0.020 ± 0.011
Erythrocytes (26453-1)	0.020 ± 0.006
Calcium.ionized [POCT] (19072-8)	0.017 ± 0.008
OPS-5-01 Incision (trepanation), excision and destruction on the skull, brain and meninges	0.017 ± 0.005
Sodium [POCT] (2951-2)	0.017 ± 0.013
Haemoglobin [POCT] (718-7)	0.017 ± 0.011
Urea (3091-6)	0.016 ± 0.008
Aspartate aminotransferase (1920-8)	0.016 ± 0.008
OPS-5-02 Other operations on skull, brain and meninges	0.015 ± 0.007

### Comparison with established risk scores

**Figure 4:**
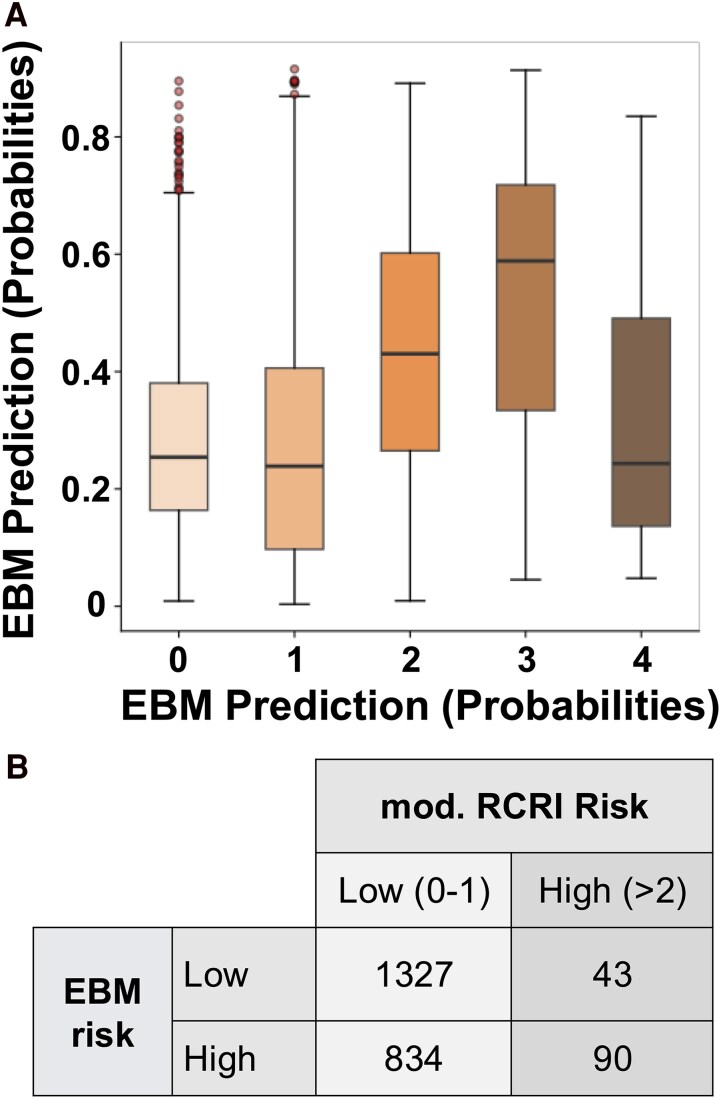
Comparison of EBM-derived risk predictions and modified revised cardiac risk Index categories. (*A*) Distribution of predicted risk probabilities from the EBM across categories of the modified RCRI. (*B*) Cross-tabulation of patients stratified into low and high risk by the EBM and the modified RCRI. The EBM identified additional high-risk patients not captured by the RCRI.

The EBM achieved markedly higher predictive accuracy than the mRCRI (*Table 2*). To explore how their predictions differ in practice, we compared individual risk estimates from both models (*Figure 4A*). EBM-predicted probabilities increased across higher RCRI categories, indicating some concordance between the models. However, substantial variability was observed within each RCRI group, reflecting additional risk factors incorporated by the EBM. Among patients classified as low risk by the RCRI (score 0–1), 834 (36.4%) were assigned to the high-risk group by the EBM. Conversely, 43patients with high RCRI scores (>2) were classified as low risk by the EBM (*Figure 4B*).

### Individual-level predictions and clinical actionability

Synthetic patient profiles were generated from the original data structure to illustrate individual-level predictions without compromising patient privacy (see Supplementary material online, *Figure S4*). Synthetic data were used because combinations of clinical variables could—with sufficient background knowledge—uniquely identify patients.

The two examples represent a low-risk and a high-risk profile with typical perioperative characteristics (see Supplementary material online, *Figure S4A*). In the low-risk example, an uncomplicated appendectomy and stable laboratory values resulted in predominantly negative feature contributions, including young age, lowering the predicted PMI risk (see Supplementary material online, *Figure S4B*). In contrast, the high-risk example combined advanced age, elevated creatine kinase, hepatic dysfunction, and respiratory failure, each contributing positively to the predicted risk (see Supplementary material online, *Figure S4C*). These examples illustrate how the EBM model aggregates multiple clinical factors into individual risk estimates.

### Clinical risk stratification compared with practice guidelines

**Figure 5:**
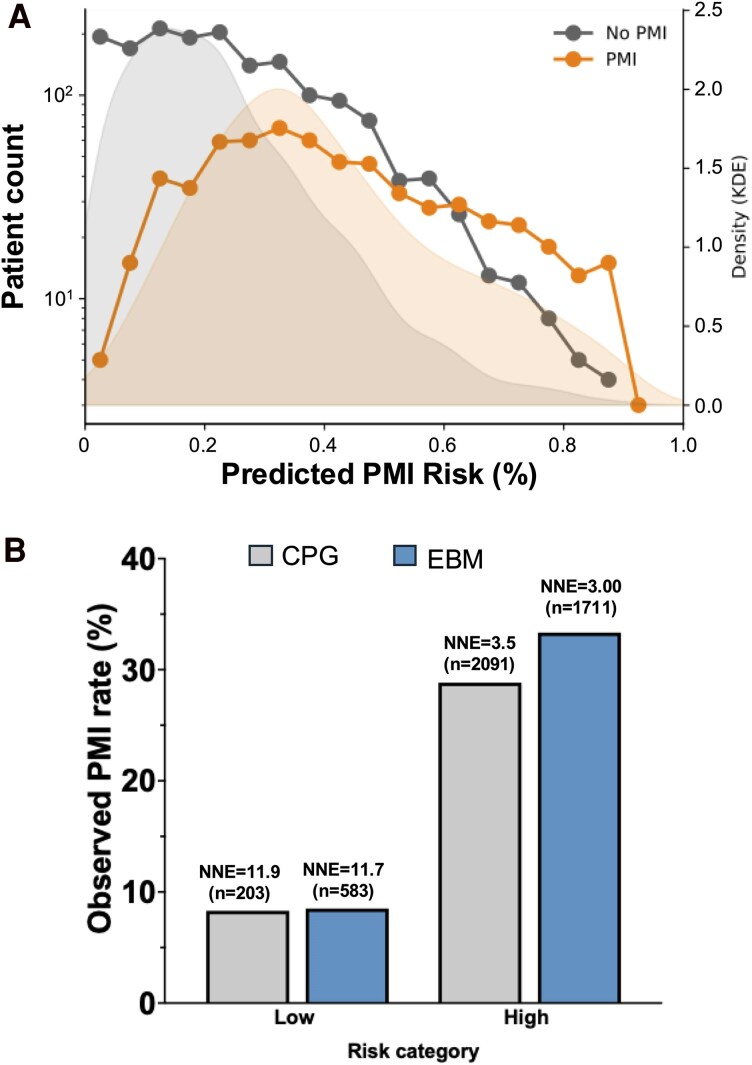
Risk distribution and stratification performance of the EBM model vs. guideline-based classification. (*A*) Distribution of patients with and without PMI across the EBM-predicted risk spectrum. Non-PMI cases (grey) cluster at the lower end of the predicted risk range, while PMI cases (orange) are increasingly enriched at higher predicted risk levels, demonstrating the discriminative capacity of the model. (*B*) Observed PMI rates and corresponding Number Needed to Evaluate (NNE) across low- and high-risk categories, comparing the EBM model (blue) with the ESC guideline-based clinical practice guideline (CPG) approach (grey). In the high-risk category, the EBM identified patients with a higher observed PMI rate (33.3%; NNE 3.00) compared with guideline-based stratification (28.6%; NNE 3.5). Values indicate observed PMI rates, NNE, and patient counts in the temporally independent validation cohort (2022–2023).

To assess clinical risk stratification, we examined the distribution of predicted PMI risk across the cohort (*Figure 5*). Patients without PMI clustered predominantly in the lower predicted risk range, while PMI cases were more evenly distributed, with progressive enrichment in the higher EBM-predicted risk strata (*Figure 5A*). When compared with the 2022 ESC Guidelines on cardiovascular assessment and management of patients undergoing non-cardiac surgery, the EBM identified a moderately larger proportion of patients as high risk and demonstrated greater enrichment of PMI events within the high-risk stratum (*Figure 5B*). The PMI incidence in the EBM-defined high-risk group was 33.3%, corresponding to a Number Needed to Evaluate (NNE) of 3.00. In comparison, the guideline-based high-risk group had a PMI incidence of 28.6%, with an NNE of 3.5.

Assuming one pre-operative and two post-operative high-sensitivity cardiac troponin determinations per high-risk patient, guideline-directed screening would have required 6273 assays in our temporally independent validation cohort (2022–2023) (2091 patients × 3 samples). An EBM-guided strategy restricted to patients classified as high risk (*n* = 1711) would have required 5133 assays (1711 × 3), corresponding to an absolute reduction of 1140 assays (18.2%). Because the EBM risk threshold is tunable, laboratory workload can be modulated: increasing the threshold further reduces assay numbers at the expense of sensitivity, whereas lowering it expands testing and case capture. This flexibility permits centres to calibrate the trade-off between resource use and diagnostic yield according to local clinical and operational priorities. Overall, these findings quantify differences in the efficiency and precision of risk identification between EBM-based and guideline-based stratification strategies.

### Deployment of EBM for clinical use

**Figure 6:**
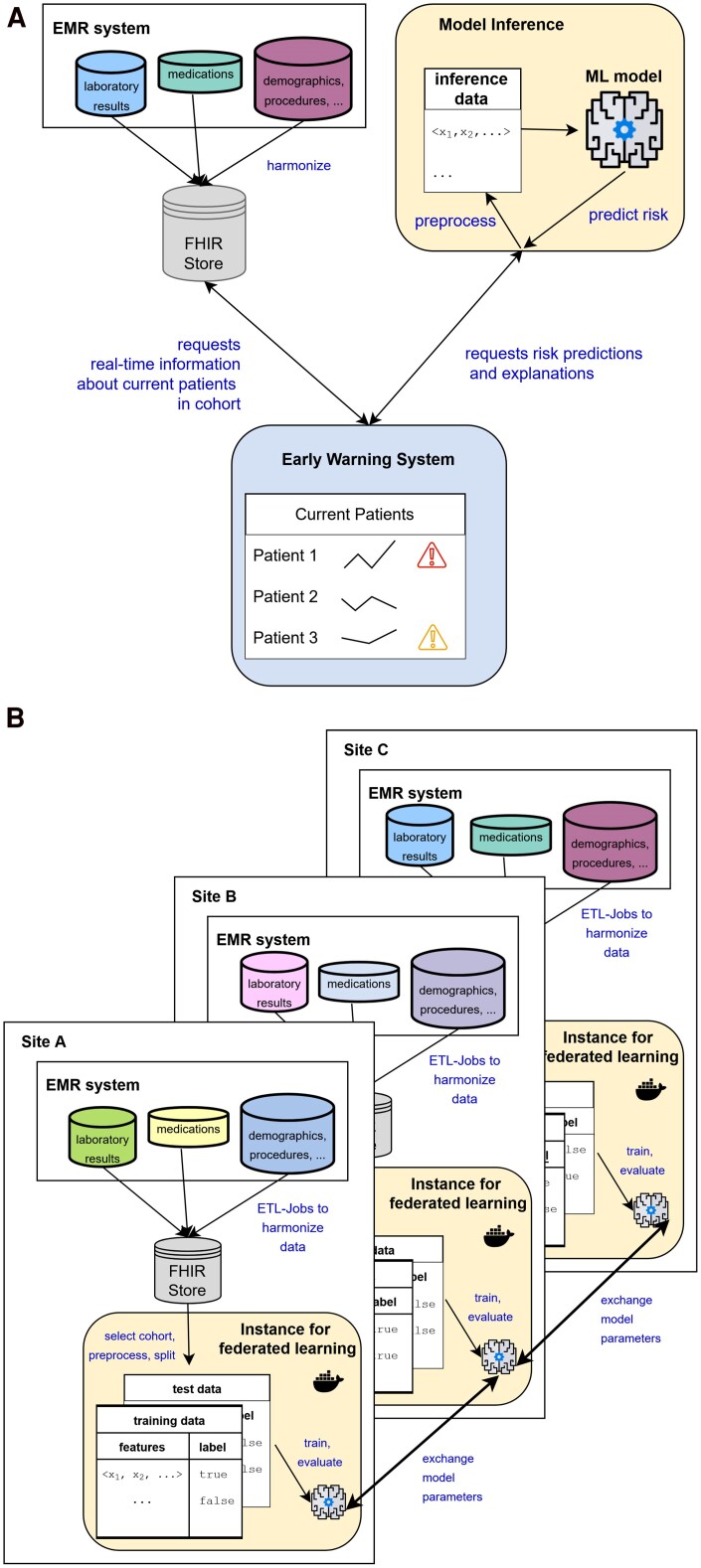
Conceptual framework for clinical integration and federated learning of the perioperative myocardial injury prediction model. (*A*) Example of real-time clinical integration using an electronic medical record (EMR) system, which harmonizes patient data into a FHIR store. The Explainable Boosting Machine model retrieves patient data, calculates perioperative myocardial injury risk, and transmits risk predictions and explanations to an early warning system for clinical monitoring. (*B*) Federated learning architecture across three independent clinical sites. Each site harmonizes local EMR data, trains a local instance of the machine learning model, and exchanges only model parameters not patient data enabling collaborative model refinement while preserving data privacy.

To support swift adoption, we designed a system integration that uses routinely collected clinical data to generate real-time risk predictions (*Figure 6*). The model infers scores automatically, without requiring manual data entry, and processes missing values through imputation or native tolerance. It can update predictions at configurable intervals, such as daily, or compute them on-demand, for instance during pre-operative assessments. To enable cross-institutional deployment, we implemented Fast Healthcare Interoperability Resources (FHIR) as a standardized data format of the medical informatics initiatives core data set and used internationally established coding systems such as LOINC, ICD-10, and OPS for laboratory values, diagnoses, and procedures. Deployment and evaluation of this system in routine clinical practice are planned as future steps.

*Figure 6A* illustrates a feasible local deployment concept. Parts of the conceptualized pipeline are already implemented locally. The electronic medical record system provides structured data on laboratory results, and procedures, which are harmonized into a local FHIR data store. An extended inference pipeline retrieves new or updated patient data, pre-processes them, and applies the machine learning model to generate risk predictions and explanatory outputs. These results feed into an early warning system that displays risk levels for current inpatients. This enables the care team to identify high-risk individuals and initiate timely interventions. The system monitors patient data continuously in the background or responds to defined clinical events.

*Figure 6B* depicts the existing federated learning framework designed for multi-site collaboration. Each institution harmonizes its routine data locally according to the CDS of the MII and trains its own instance of the model. Rather than exchanging patient-level data, sites share only model parameters. A central server aggregates these parameters and redistributes updated weights, allowing collaborative model refinement while protecting data privacy and institutional boundaries.

To accommodate differences in data distributions or patient populations between sites, institutions can fine-tune model parameters locally before clinical deployment.

## Discussion

PMI remains a frequent and underrecognized complication of non-cardiac surgery, with substantial implications for patient outcomes and perioperative care. In this study, we developed and evaluated an interpretable machine learning model using the EBM to improve risk prediction based solely on routine data. The EBM outperformed conventional clinical risk scores, combining high discriminative accuracy with robust calibration across risk strata, while preserving full model transparency. Feature analysis showed that both acute perioperative factors and chronic comorbidities were key drivers of PMI risk. Compared with guideline-based stratification, the EBM modestly improved risk enrichment and reduced projected troponin measurements by 18% in the temporally independent validation cohort.

Troponin testing in our cohort was performed according to clinical judgment rather than systematic universal screening. This reflects real-world practice but introduces potential selection bias, as higher-risk patients are more likely to undergo biomarker assessment. We therefore explicitly compared tested and untested patients and evaluated model discrimination and calibration within the subgroup of patients with available troponin measurements. Importantly, calibration remained stable across predicted risk strata, suggesting that the model captured clinically meaningful risk signals rather than merely reproducing physician testing behaviour. Nevertheless, the absence of systematic screening may have led to underestimation of the absolute PMI incidence and could attenuate performance metrics. Importantly, the present model should not be interpreted as a universal screening instrument. Rather, it estimates PMI risk conditional on a testing-enriched population in whom post-operative troponin assessment is considered or recommended. This selection mechanism may influence the distribution and apparent importance of predictors and could affect calibration when applied to unselected low-risk surgical populations. Accordingly, we frame our findings as most applicable to risk-guided perioperative surveillance pathways, with external validation required before broader generalization. Our approach relies on routinely collected, harmonized data, allowing the model to be fine-tuned and deployed at other institutions for automated risk stratification.

Several previous studies have applied machine learning to predict PMI. Compared with the XGBoost-based approach by Oh *et al*.^34^ our study differs in methodological design and clinical focus. Although their model achieved reasonable performance (AUC 0.78) using a combination of pre-operative and intraoperative data, it was limited to patients with normal pre-operative troponin levels, which constrains its generalizability. Although the EBM yielded slightly lower discriminative accuracy (AUC 0.73), it demonstrated superior calibration and substantially greater interpretability. Its additive structure, based on non-linear shape functions for each predictor, enables visualization of individual risk contributions and supports transparent reasoning. This is essential for clinical trust and real-world deployment.

Traditional risk scores, such as the RCRI and the NSQIP-based MICA calculator, rely on a small number of variables, limiting scalability and predictive performance. These models frequently underperform in high-risk populations and often exhibit poor calibration. In an external validation cohort undergoing vascular surgery, both the RCRI and MICA demonstrated limited discrimination (AUC 0.60 and 0.64, respectively) and consistently underestimated the risk of adverse events.^6,35^ These limitations are also reflected in real-world practice. A recent multi-centre survey found that fewer than 15% of academic surgeons reported using the NSQIP calculator in more than 40% of pre-operative evaluations, citing limited integration, insufficient usefulness, and inadequate performance in high-risk cases.^36^ In contrast, our EBM model incorporated a broader set of routinely available features to generate dynamic, individualized risk predictions. This resulted in improved accuracy, stronger calibration, and significantly greater efficiency in identifying high-risk patients, as demonstrated by the lower number needed to evaluate. The EBM therefore represents a scalable and clinically meaningful complement to existing risk prediction systems, enabling automated, data-driven pre-screening within routine workflows.

All model inputs were derived from structured data routinely collected in German hospitals, facilitating technical integration into electronic health record systems and pre-operative workflows. In contrast to guideline-based strategies that rely on categorical risk criteria and fixed thresholds, our model generates continuous, individualized risk estimates that are directly actionable and support patient-specific surveillance strategies. Although current ESC guidelines recommend perioperative troponin testing in patients at elevated cardiovascular risk, real-world adherence remains low.^9,37^ Against this backdrop, our model offers a data-driven mechanism to operationalize guideline recommendations by identifying those patients most likely to benefit from targeted testing and monitoring. Notably, in our hold-out test cohort, strict adherence to ESC-based screening would have required more than 6200 individual troponin measurements. By contrast, our model enables automated risk stratification using routinely available data and supports selective biomarker testing in patients at highest predicted risk. These clinical utility estimates are exploratory and do not define actionable thresholds. Because the EBM risk threshold is tunable, institutions can modulate the trade-off between testing volume and case capture according to local clinical and operational priorities; prospective interventional studies are required to determine whether EBM-guided surveillance improves outcomes or resource utilization. This approach may enhance clinical efficiency and resource allocation, particularly given that only 6.8% of guideline-eligible patients received pre-operative biomarker screening in a recent multicentre study of non-cardiac surgical patients.^9^

In addition, the EBM provides interpretable, patient-specific predictions that enable clinicians to identify clinically meaningful and potentially modifiable risk factors.^14,15^ In our cohort, such features included renal impairment, anaemia, and elevated creatine kinase. This local interpretability allows for actionable insights at the individual level, which is essential for personalized medicine. Such insight supports individualized management, including enhanced monitoring or specialist involvement. Similar benefits have been demonstrated in cardiovascular risk prediction using proteomic data, where EBM models not only outperformed conventional risk scores but also clarified how individual biomarkers contributed to predicted risk across diverse patient subgroups.^38^

Transparent models like EBM support clinical trust and help uncover underlying pathophysiological mechanisms and therapeutic targets-capabilities that black-box models cannot provide.^39,40^ Pre-operative risk stratification may guide downstream decisions about telemetry, ICU admission, or mobilization, improving resource allocation and efficiency.^39,41^ The ability to stratify risk at the patient level, coupled with model interpretability, provides a strong foundation for individualized care, targeted interventional studies, and continuous model refinement based on clinical feedback.^42^

Beyond risk prediction, perioperative myocardial injury is increasingly understood within a broader framework of inflammation, hypoxia signalling, and microvascular dysfunction. Translational strategies targeting hypoxia-inducible factor (HIF) pathways have demonstrated cardioprotective and organ-protective effects in inflammatory and ischaemic injury models.^43^ Similarly, extracellular adenosine signalling has been identified as a central adaptive mechanism attenuating ischemia–reperfusion injury and inflammatory tissue damage.^44^ In parallel, microRNA-mediated regulatory mechanisms have emerged as modulators of perioperative organ injury and potential therapeutic targets.^45^ While our study does not directly interrogate these pathways, interpretable risk models such as the EBM may facilitate patient enrichment and trial design for future mechanistically targeted interventions by identifying individuals at highest biological risk.

All predictor variables were strictly restricted to information documented before the day of surgery. ICD-10 diagnoses recorded after the index procedure and post-operative complications were excluded, OPS codes were used to characterize the planned surgical procedure, and laboratory values were limited to pre-defined pre-operative windows. No intraoperative, post-operative, or outcome-related variables were accessible during model training or validation, reducing the risk of temporal leakage.

All variables used in model training were derived from structured clinical documentation. The widespread adoption of the FHIR standard across German university hospitals provides a strong foundation for technical deployment.^26,46,47^ We embedded the EBM into a clinical decision support architecture capable of real-time inference, integration into pre-operative checklists, and continuous early warning systems. Its additive and interpretable structure not only meets regulatory expectations but also enables clinicians to understand the drivers of each individual risk estimate, thereby supporting trust and targeted risk mitigation. The model can be fine-tuned on local data to reflect institutional case mix and deployed across sites using federated learning frameworks, which preserve data privacy by sharing only model parameters rather than patient-level data.^46^

Our study has several important strengths. It is based on a large, clinically heterogeneous cohort enriched through guideline-directed post-operative troponin testing at a tertiary care centre in Germany. The model was benchmarked against established clinical reference standards, including the RCRI and the ESC-guideline-based troponin screening strategy, and evaluated using a temporally independent validation strategy. In these comparisons, the EBM demonstrated equal or superior performance, combining accurate risk prediction with strong calibration and full interpretability

Several limitations should also be acknowledged. First, this was a retrospective single-centre study, which may limit generalizability and carries an inherent risk of residual confounding.^34^ Second, troponin measurements were not obtained according to a standardized screening protocol but were ordered based on clinical judgment. This may have introduced selection bias and could lead to underestimation of the true incidence of PMI. Third, external validation in independent clinical populations and diverse institutional settings is required to confirm model portability and calibration stability. Fourth, the accuracy and consistency of administrative coding (e.g. ICD and OPS codes) may vary between hospitals, potentially affecting the reliability of input features and limiting scalability.^48^ Fifth, although we applied uniform winsorization and model-specific missing-data handling, alternative pre-processing choices (e.g. different outlier handling or imputation strategies) could influence model behaviour; robustness under alternative pre-processing pipelines should be examined in future validation studies. Finally, while the model demonstrated strong retrospective performance, its prospective impact on clinical workflows and patient outcomes has yet to be evaluated. Future studies should assess the feasibility and effectiveness of deploying this model in real-time perioperative settings.

In summary, our study demonstrates that an interpretable machine learning model can accurately predict perioperative myocardial injury using routinely collected pre-operative data. The EBM offers strong discriminative performance, robust calibration, and clinical transparency. By supporting automated, individualized risk stratification, it provides a scalable foundation for improving perioperative decision-making, targeted surveillance, and efficient resource allocation. Prospective validation in diverse clinical environments is now warranted to assess its real-world impact and integration into perioperative care pathways.

## Supplementary Material

ztag093_Supplementary_Data

## Data Availability

The individual-level clinical data used in this study contain sensitive health information and therefore cannot be made publicly available due to data protection regulations. A *comparable* dataset based on routine care data from German university hospitals can be requested via the German national health data research platform, the Forschungsdatenportal Gesundheit (FDPG, https://fdpg.de), subject to approval by the responsible data access bodies. The code used for model development and evaluation is openly available at https://github.com/meDIC-UKT/PeriMyo. Further information is available from the corresponding author upon reasonable request.
