# Supplementary material for: Interpretable machine learning models for predicting perioperative myocardial injury in non-cardiac surgery
Source: Eur Heart J Digit Health. 2026 Jun 12;7(6):ztag093. doi: 10.1093/ehjdh/ztag093 (PMC13310016; doi:10.1093/ehjdh/ztag093)
Supplement: ztag093_Supplementary_Data [file ztag093_supplementary_data.zip › !04 - Supplemental - FIG - 241119 MS EBM in PMI_revised.pdf]

Supplemental Figure 1

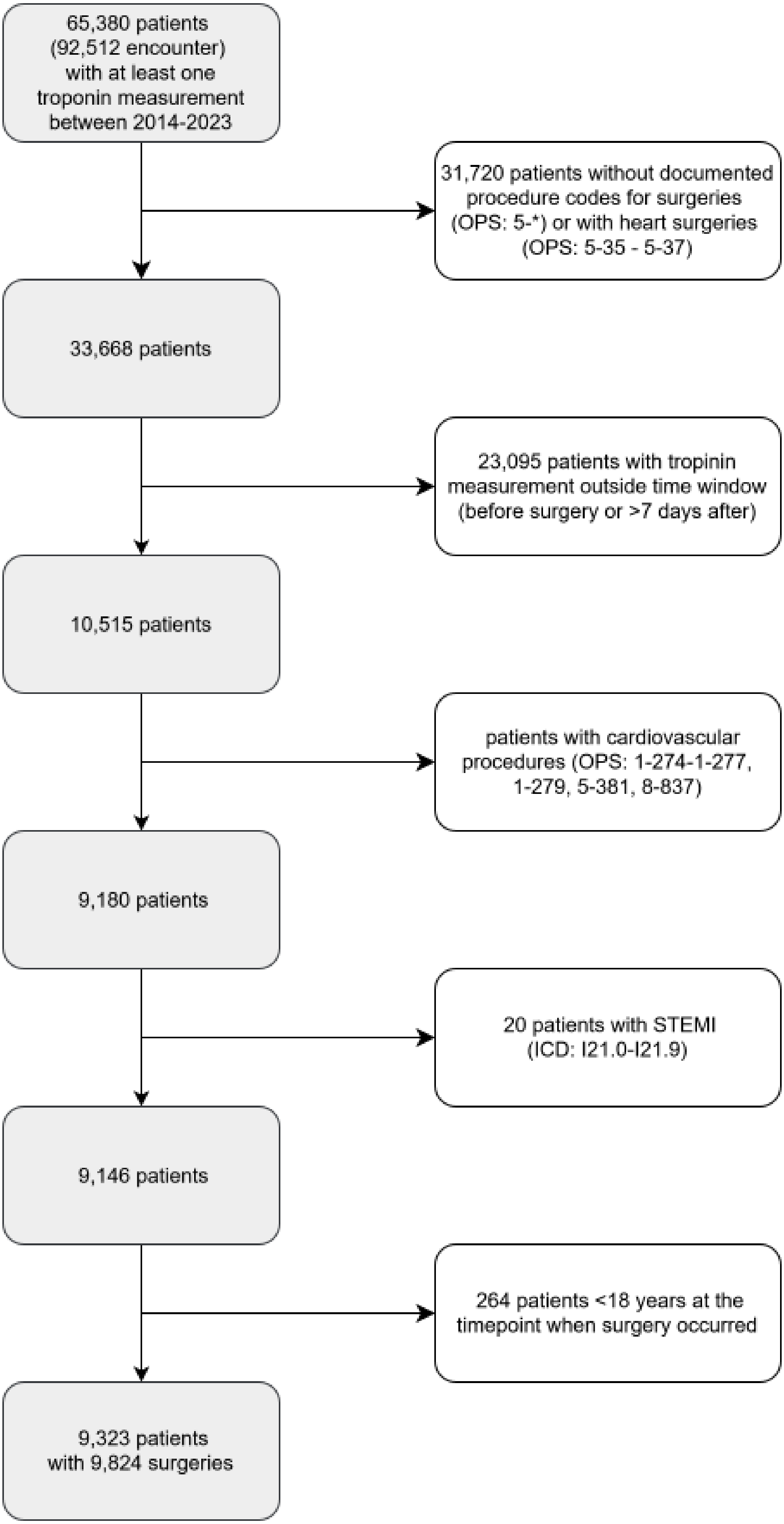

Supplemental Figure 1. Study flow diagram for cohort selection. Flowchart illustrating patient selection for the final analytic cohort. Between 2014 and 2023, 65,380 patients (92,512 surgical encounters) with at least one troponin measurement were screened. Patients without documented surgical procedure codes (OPS 5-*) or with cardiac surgery codes (OPS 5-35–5-37), troponin measurements outside the predefined perioperative time window (before surgery or >7 days postoperatively), cardiovascular procedures (OPS 1-274–1-277, 1-279, 5-381, 8-837), ST-elevation myocardial infarction (ICD-10 I21.0–I21.9), or age <18 years at the time of surgery were excluded. The final study cohort comprised 9,323 adult patients undergoing 9,824 non-cardiac surgical procedures.

Supplemental Figure 2

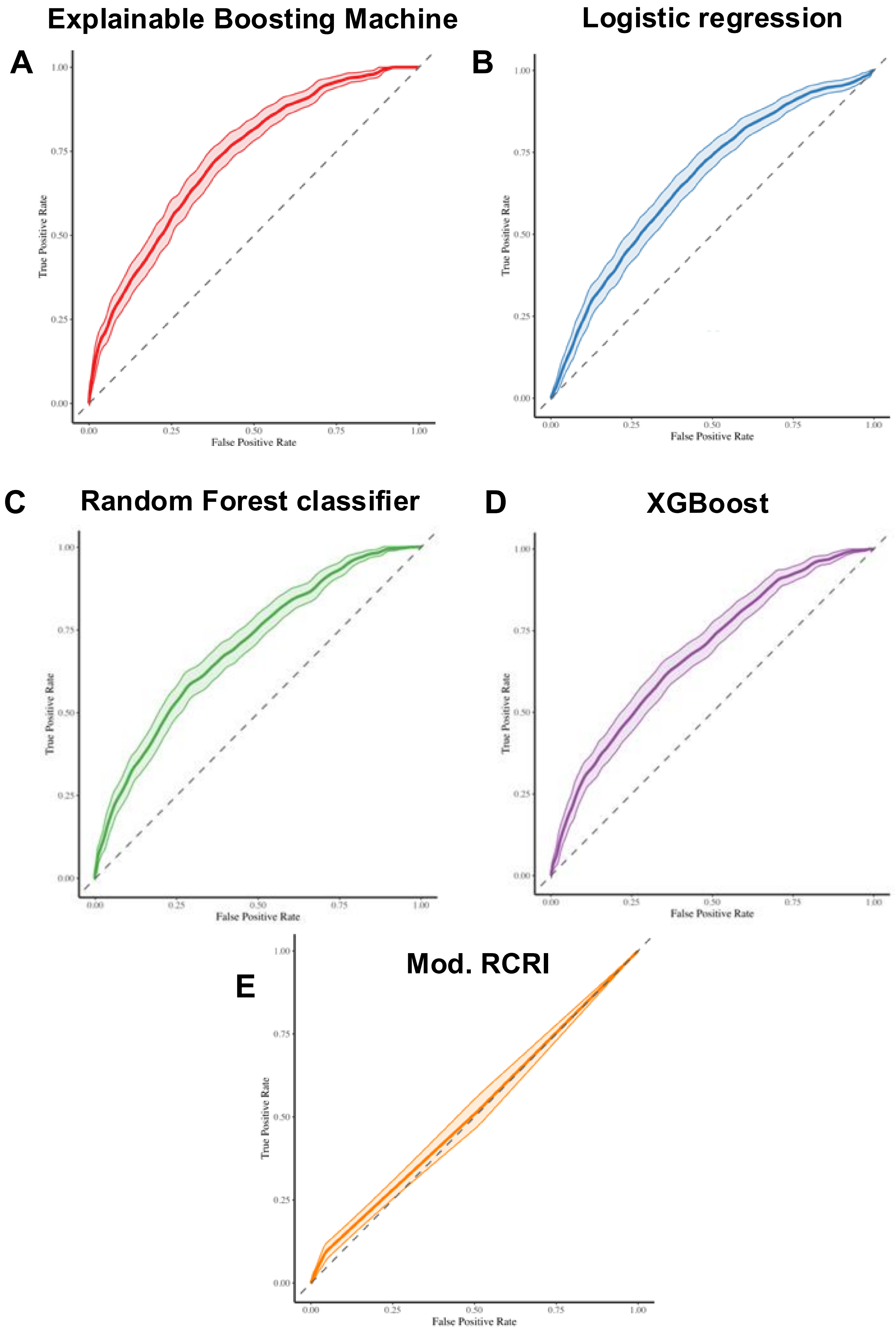

Supplemental Figure 2. Receiver operating characteristic ROC curves for machine learning models and the modified Revised Cardiac Risk Index.

Receiver operating characteristic ROC curves are shown for the Explainable Boosting Machine in panel **A**, logistic regression in panel **B**, random forest classifier in panel **C**, XGBoost in panel **D**, and the modified Revised Cardiac Risk Index Mod. RCRI in panel E in the independent test cohort. Solid lines represent the mean ROC curve across five cross validation repetitions. Shaded areas indicate bootstrapped 95 percent confidence intervals. The diagonal dashed line represents no discrimination.

Suppl. Fig. 3

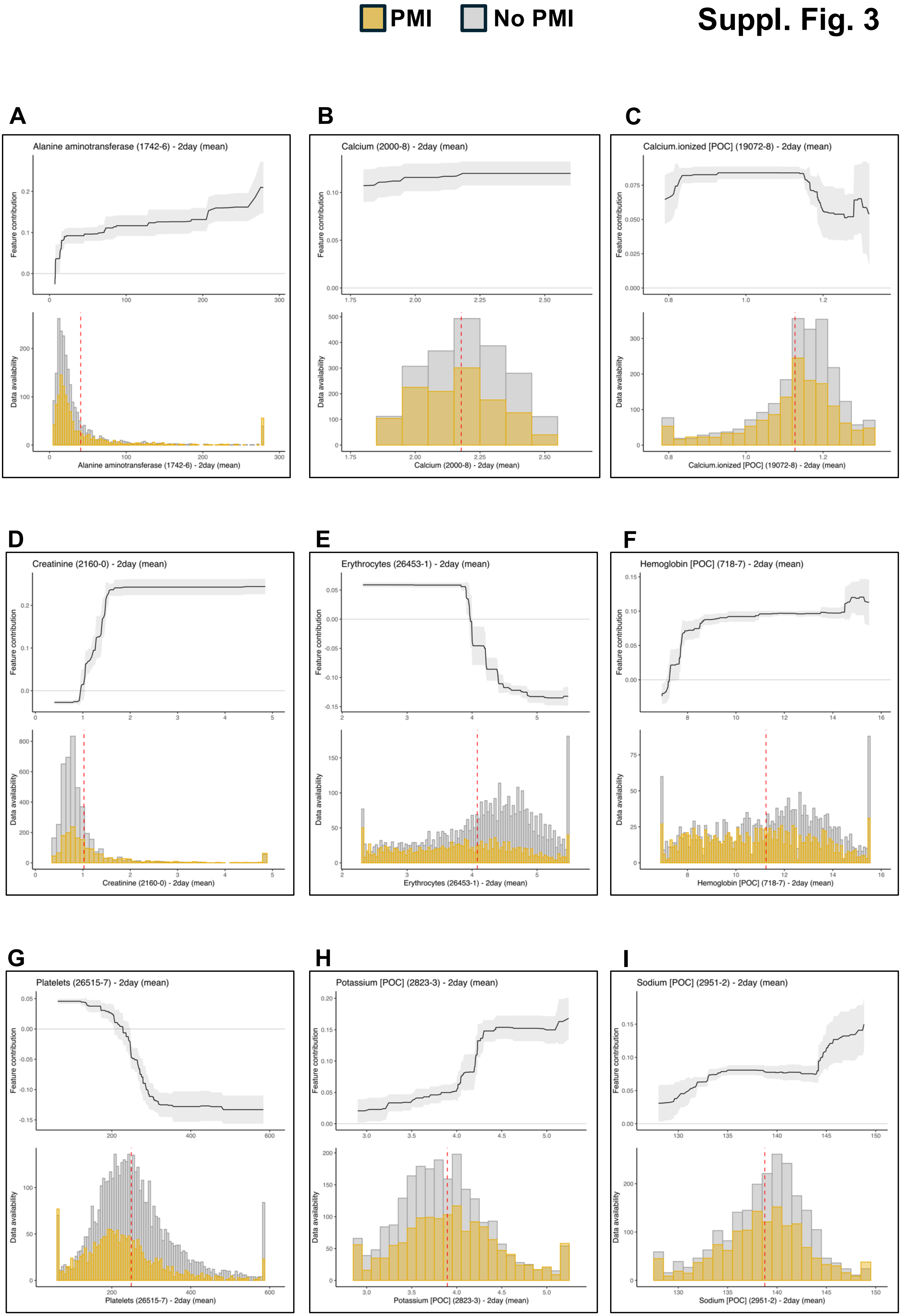
PMI
No PMI
A
Alanine aminotransferase (1742-6) - 2day (mean)
Feature contribution
Data availability
Alanine aminotransferase (1742-6) - 2day (mean)
B
Calcium (2000-8) - 2day (mean)
Feature contribution
Data availability
Calcium (2000-8) - 2day (mean)
C
Calcium.ionized [POC] (19072-8) - 2day (mean)
Feature contribution
Data availability
Calcium.ionized [POC] (19072-8) - 2day (mean)
D
Creatinine (2160-0) - 2day (mean)
Feature contribution
Data availability
Creatinine (2160-0) - 2day (mean)
E
Erythrocytes (26453-1) - 2day (mean)
Feature contribution
Data availability
Erythrocytes (26453-1) - 2day (mean)
F
Hemoglobin [POC] (718-7) - 2day (mean)
Feature contribution
Data availability
Hemoglobin [POC] (718-7) - 2day (mean)
G
Platelets (26515-7) - 2day (mean)
Feature contribution
Data availability
Platelets (26515-7) - 2day (mean)
H
Potassium [POC] (2823-3) - 2day (mean)
Feature contribution
Data availability
Potassium [POC] (2823-3) - 2day (mean)
I
Sodium [POC] (2951-2) - 2day (mean)
Feature contribution
Data availability
Sodium [POC] (2951-2) - 2day (mean)

Supplemental Figure 3. Feature contributions and distributions for selected preoperative laboratory variables in perioperative myocardial injury prediction.

Panels A–I display marginal effect plots and corresponding value distributions for selected laboratory predictors identified by the Explainable Boosting Machine (EBM): alanine aminotransferase (A), total calcium (B), ionized calcium [POC] (C), creatinine (D), erythrocytes (E), hemoglobin [POC] (F), platelets (G), potassium [POC] (H), and sodium [POC] (I).

For each variable, the upper panel illustrates the marginal contribution to the log-odds of predicted perioperative myocardial injury, with shaded areas representing 95% confidence intervals. The lower panel shows the distribution of preoperative laboratory values in patients with PMI (yellow) and without PMI (grey). Red dashed vertical lines indicate the median value in the overall cohort. Laboratory values represent the mean measurement obtained within 2 days prior to surgery. Laboratory identifiers in parentheses correspond to *Logical Observation Identifiers Names and Codes* (LOINC)

Supplemental Figure 4

A

Variable	Low Risk	High-Risk
Age	35	69
Sex	Male	Male
Charlson Index	0	0
eGFR (ml/min/m²)	93.8	17.5
Hemoglobin (g/dl)	12.9	8.7
Erythrocytes (Mio/µl)	4.3	3.0
Vascular procedures	Absent	Present

B

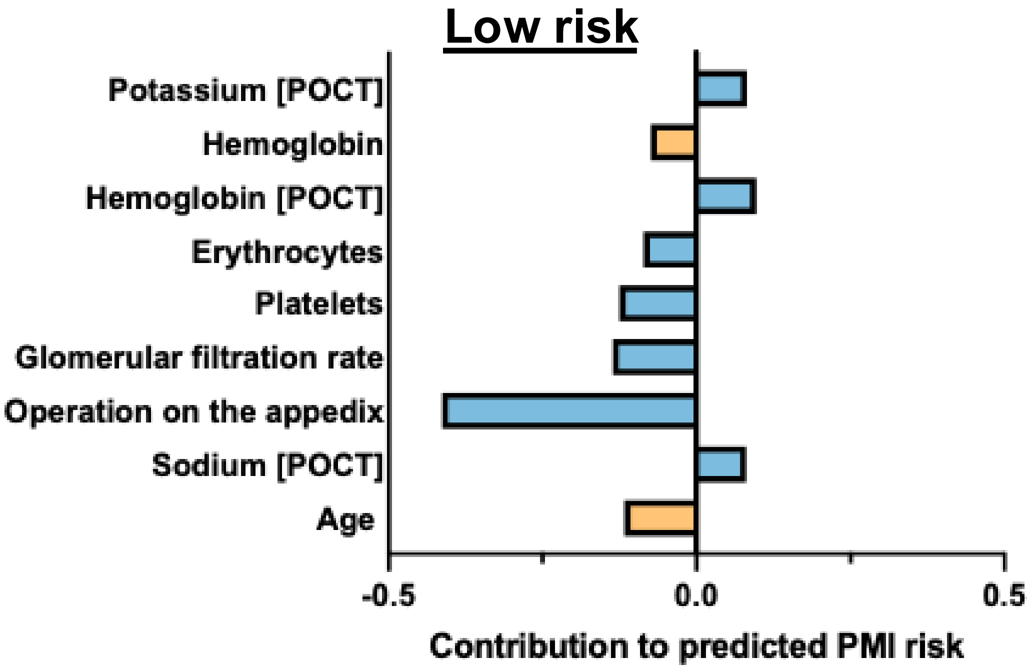

C

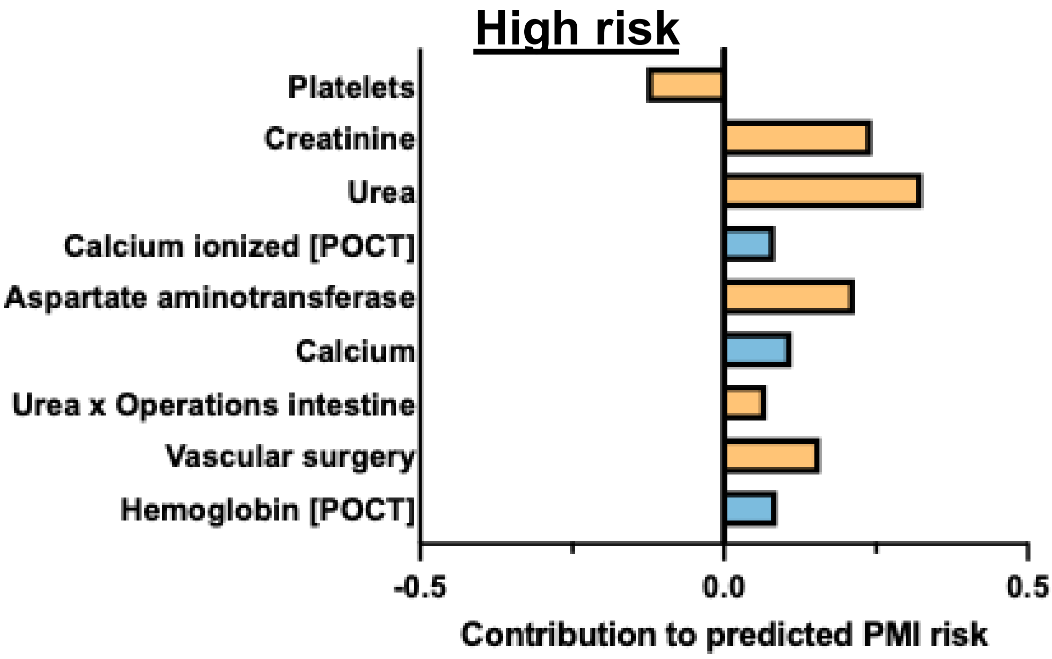

Supplemental Figure 4. Illustrative patient profiles and individual feature contributions for low- and high-risk predictions.

(A) Example patient characteristics for a low risk and a high risk case as predicted by the Explainable Boosting Machine EBM model. The high risk example is characterized by older age, markedly impaired renal function reflected by reduced eGFR, lower hemoglobin and erythrocyte levels, and the presence of vascular procedures, whereas the low risk example shows preserved renal function and absence of vascular surgery. The Charlson comorbidity index was zero in both cases.

(B, C) Individual feature contributions to predicted perioperative myocardial injury PMI risk for the low risk case in panel B and the high risk case in panel C. Bars represent additive contributions on the log odds scale relative to the model intercept. In the low risk case most features contribute to a reduction in predicted PMI risk. In the high risk case impaired renal function reflected by creatinine and urea, liver associated laboratory abnormalities, vascular surgery, and selected interaction terms contribute positively to the predicted PMI risk, while some hematologic parameters exert mitigating effects.
