# Supplementary material for: Interpretable machine learning models for predicting perioperative myocardial injury in non-cardiac surgery
Source: Eur Heart J Digit Health. 2026 Jun 12;7(6):ztag093. doi: 10.1093/ehjdh/ztag093 (PMC13310016; doi:10.1093/ehjdh/ztag093)
Supplement: ztag093_Supplementary_Data [file ztag093_supplementary_data.zip › !05 - Supplemental Table_Revision.pdf]

Supplemental Table 1 – OPS mortality risk

OPS code	Procedure Description	Total cases	In-hospital Deaths, n (%)	Surgery Risk
OPS-5-01	Incision (trepanation), excision and destruction on the skull, brain and meninges	651.859	64,210 (9.85%)	high
OPS-5-02	Other operations on skull, brain and meninges	419.028	46,198 (11.03%)	high
OPS-5-03	Operations on spinal cord, spinal cord membranes and spinal canal	2.433.378	28,041 (1.15%)	medium
OPS-5-04	Operations on nerves and nerve ganglia	103.533	212 (0.2%)	low
OPS-5-05	Other operations on nerves and nerve ganglia	319.593	1,219 (0.38%)	low
OPS-5-06	Operations on thyroid gland and parathyroid gland	645.197	1,161 (0.18%)	low
OPS-5-07	Operations on other endocrine glands	34.215	419 (1.22%)	medium
OPS-5-08	Operations on lacrimal gland and lacrimal ducts	33.491	0 (0.0%)	low
OPS-5-09	Operations on the eyelids	258.742	57 (0.02%)	low
OPS-5-10	Operations on the eye muscles	51.941	0 (0.0%)	low
OPS-5-11	Operations on the conjunctiva	47.747	23 (0.05%)	low
OPS-5-12	Operations on the cornea	157.854	48 (0.03%)	low
OPS-5-13	Operations on iris, ciliary body, anterior eye chamber, and sclera	552.822	140 (0.03%)	low
OPS-5-14	Operations on the lens	626.096	66 (0.01%)	low
OPS-5-15	Operations on retina, choroidea and vitreous body	1.283.181	273 (0.02%)	low
OPS-5-16	Operations on orbit and eyeball	59.014	52 (0.09%)	low
OPS-5-18	Operations on auricle and external auditory canal	222.464	203 (0.09%)	low
OPS-5-19	Microsurgical operations on the middle ear	133.002	0 (0.0%)	low
OPS-5-20	Other operations on middle and inner ear	331.948	477 (0.14%)	low
OPS-5-21	Operations on the nose	1.369.452	1,515 (0.11%)	low
OPS-5-22	Operations on the paranasal sinuses	445.219	489 (0.11%)	low
OPS-5-23	Removal and restoration of teeth	219.178	2,044 (0.93%)	low
OPS-5-24	Operations on gums, alveolae and jaw	155.470	913 (0.59%)	low

OPS-5-25	Operations on the tongue	49.227	252 (0.51%)	low
OPS-5-26	Operations on salivary glands and salivary gland excretory ducts	100.334	136 (0.14%)	low
OPS-5-27	Other operations on mouth and face	157.134	1,154 (0.73%)	low
OPS-5-28	Operations in the nasopharyngeal and oropharyngeal area	495.625	493 (0.1%)	low
OPS-5-29	Surgery on the pharynx	55.553	355 (0.64%)	low
OPS-5-30	Larynx excision and resection	98.987	212 (0.21%)	low
OPS-5-31	Other larynx surgery and surgery on the trachea	303.227	54,269 (17.9%)	high
OPS-5-32	Excision and resection in lung and bronchus	196.047	7,168 (3.66%)	medium
OPS-5-33	Other operations on lungs and bronchus	138.955	9,524 (6.85%)	high
OPS-5-34	Operations on chest wall, pleura, mediastinum and diaphragm	476.505	45,828 (9.62%)	high
OPS-5-35	Operations on cardiac valves and septa and vessels near the heart	546.695	22,168 (4.05%)	medium
OPS-5-36	Operations on the coronary vessels	337.809	15,310 (4.53%)	medium
OPS-5-37	Rhythm surgery and other operations on heart and pericardium	1.030.930	39,438 (3.83%)	medium
OPS-5-38	Incision, excision and closure of blood vessels	1.954.295	90,361 (4.62%)	medium
OPS-5-39	Other operations on blood vessels and additional information about operations on blood vessels	1.352.488	66,614 (4.93%)	medium
OPS-5-40	Operations on lymph tissue	859.082	9,036 (1.05%)	medium
OPS-5-41	Operations on spleen and bone marrow	67.786	5,353 (7.9%)	high
OPS-5-42	Operations on the oesophagus	361.305	21,680 (6.0%)	high
OPS-5-43	Incision, excision and resection of stomach	462.203	31,667 (6.85%)	high
OPS-5-44	Other operations on the stomach	435.952	32,004 (7.34%)	high
OPS-5-45	Incision, excision, resection and anastomosis of small and large intestine	1.731.347	83,961 (4.85%)	medium
OPS-5-46	Other operations on small and large intestine	2.358.858	132,334 (5.61%)	high
OPS-5-47	Operations on the appendix	558.788	5,143 (0.92%)	low
OPS-5-48	Operations on the rectum	363.337	9,338 (2.57%)	medium
OPS-5-49	Operations on the anus	705.327	2,219 (0.31%)	low
OPS-5-50	Operations on the liver	135.558	9,927 (7.32%)	high
OPS-5-51	Operations on gallbladder and bile ducts	2.489.118	81,588 (3.28%)	medium

OPS-5-52	Operations on the pancreas	195.275	12,414 (6.36%)	high
OPS-5-53	Closure of abdominal hernias	1.461.945	9,797 (0.67%)	low
OPS-5-54	Other operations in the abdominal area	978.777	114,644 (11.71%)	high
OPS-5-55	Operations on the kidney	465.630	7,800 (1.68%)	medium
OPS-5-56	Operations on the ureter	775.739	6,553 (0.84%)	low
OPS-5-57	Operations on the bladder	1.197.319	20,391 (1.7%)	medium
OPS-5-58	Operations on the urethra	193.051	618 (0.32%)	low
OPS-5-59	Other operations on the urinary organs	130.179	2,560 (1.97%)	medium
OPS-5-60	Operations on prostate and seminal vesicles	594.655	1,872 (0.31%)	low
OPS-5-61	Operations on scrotum and tunica vaginalis testis	77.307	558 (0.72%)	low
OPS-5-62	Operations on the testicle	147.184	696 (0.47%)	low
OPS-5-63	Operations on funiculus spermaticus, epididymis and ductus deferens	89.926	20 (0.02%)	low
OPS-5-64	Operations on the penis	168.793	731 (0.43%)	low
OPS-5-65	Operations on the ovary	724.789	2,016 (0.28%)	low
OPS-5-66	Operations on the fallopian tube	420.232	110 (0.03%)	low
OPS-5-67	Operations on the cervix uteri	99.478	44 (0.04%)	low
OPS-5-68	Incision, excision and extirpation of the uterus	699.386	1,392 (0.2%)	low
OPS-5-69	Other operations on the uterus and operations on the parametria	225.095	153 (0.07%)	low
OPS-5-70	Operations on vagina and pouch of Douglas	585.414	509 (0.09%)	low
OPS-5-71	Operations on the vulva	106.618	240 (0.23%)	low
OPS-5-72	Breech delivery and instrumental delivery	275.894	0 (0.0%)	low
OPS-5-73	Other operations for induction of labor and during birth	652.081	11 (0.0%)	low
OPS-5-74	Caesarean section and child development	1.609.752	132 (0.01%)	low
OPS-5-75	Other birth-assisting operations	1.998.974	12 (0.0%)	low
OPS-5-76	Operations on facial fractures	127.785	314 (0.25%)	low
OPS-5-77	Other operations on facial bones	209.233	741 (0.35%)	low
OPS-5-78	Operations on other bones	3.253.355	22,562 (0.69%)	low

OPS-5-79	Repositioning of fracture and luxation	3.167.890	49,424 (1.56%)	medium
OPS-5-80	Open surgical and other joint operations	1.563.472	13,881 (0.89%)	low
OPS-5-81	Arthroscopic joint operations	3.227.306	3,444 (0.11%)	low
OPS-5-82	Endoprosthetic joint and bone replacement	3.040.695	40,828 (1.34%)	medium
OPS-5-83	Operations on the spine	4.135.878	40,757 (0.99%)	low
OPS-5-84	Operations on the hand	492.629	1,731 (0.35%)	low
OPS-5-85	Operations on muscles, tendons, fasciae and bursas	1.380.184	37,933 (2.75%)	medium
OPS-5-86	Replantation, exarticulation and amputation of extremities and other operations on the locomotor system	914.253	32,700 (3.58%)	medium
OPS-5-87	Excision and resection of the mamma	545.275	195 (0.04%)	low
OPS-5-88	Other operations on the mamma	234.562	61 (0.03%)	low
OPS-5-89	Operations on skin and hypoderm	3.068.652	80,777 (2.63%)	medium
OPS-5-90	Surgical restoration and reconstruction of skin and hypoderm	1.698.722	27,190 (1.6%)	medium
OPS-5-91	Other operations on skin and hypoderm	1.137.683	61,476 (5.4%)	high
OPS-5-92	Operations on skin and hypoderm in burns and chemical burns	186.156	7,643 (4.11%)	medium
OPS-5-93	Information about the graft and the materials used	2.226.706	33,899 (1.52%)	medium
OPS-5-98	Special surgical techniques and operations in special care situations	5.691.395	80,815 (1.42%)	medium
OPS-5-99	Premature abortion of an operation	113.220	8,541 (7.54%)	high

Supplemental Table 2 – Modified Charlson Comorbidity Index

CCI subsystem	ICD10-Codes	Weight
Acute myocardial infarction	ICD-I21.x', 'ICD-I22.x', 'ICD-I25.2'	0
Congestive heart failure	ICD-I09.9', 'ICD-I11.0', 'ICD-I13.0', 'ICD-I13.2', 'ICD-I25.5', 'ICD-I42.0', 'ICD-I42.5-ICD-I42.9', 'ICD-I43.x', 'ICD-I50.x', 'ICD-P29.0'	0
Peripheral vascular disease	ICD-I70.x', 'ICD-I71.x', 'ICD-I73.1', 'ICD-I73.8', 'ICD-I73.9', 'ICD-I77.1', 'ICD-I79.0', 'ICD-I179.2', 'ICD-K55.1', 'ICD-K55.8', 'ICD-K55.9', 'ICD-Z95.8', 'ICD-Z95.9'	1
Cerebral vascular accident	ICD-G45.x', 'ICD-G46.x', 'ICD-H34.0', 'ICD-I60.x-ICD-I69.x'	1
Dementia	ICD-F00.x-ICD-F03.x', 'ICD-F05.1', 'ICD-G30.x', 'ICD-G31.1'	1
Chronic Pulmonary disease	ICD-I27.8', 'ICD-I27.9', 'ICD-J40.x-ICD-J47.x', 'ICD-J60.x-ICD-J67.x', 'ICD-J68.4', 'ICD-J70.1', 'ICD-J70.3'	1
Rheumatic disease	ICD-M05.x', 'ICD-M06.x', 'ICD-M31.5', 'ICD-M32.x-ICD-M34.x', 'ICD-M35.1', 'ICD-M35.3', 'ICD-M36.0'	1

Peptic ulcer	ICD-K25.x-ICD-K28.x'	1
Mild Liver disease	ICD-B18.x', 'ICD-K70.0-ICD-K70.3', 'ICD-K70.9', 'ICD-K71.3-ICD-K71.5', 'ICD-K71.7', 'ICD-K73.x', 'ICD-K74.x', 'ICD-K76.0', 'ICD-K76.2-ICD-K76.4', 'ICD-K76.8', 'ICD-K76.9', 'ICD-Z94.4'	1
Diabetes without chronic complication	ICD-E10.0', 'ICD-E10.1', 'ICD-E10.6', 'ICD-E10.8', 'ICD-E10.9', 'ICD-E11.0', 'ICD-E11.1', 'ICD-E11.6', 'ICD-E11.8', 'ICD-E11.9', 'ICD-E12.0', 'ICD-E12.1', 'ICD-E12.6', 'ICD-E12.8', 'ICD-E12.9', 'ICD-E13.0', 'ICD-E13.1', 'ICD-E13.6', 'ICD-E13.8', 'ICD-E13.9', 'ICD-E14.0', 'ICD-E14.1', 'ICD-E14.6', 'ICD-E14.8', 'ICD-E14.9'	1
Diabetes with chronic complication	ICD-E10.2-ICD-E10.5', 'ICD-El0.7', 'ICD-E11.2-ICD-E11.5', 'ICD-E11.7', 'ICD-E12.2-ICD-E12.5', 'ICD-E12.7', 'ICD-E13.2-ICD-E13.5', 'ICD-E13.7', 'ICD-E14.2-ICD-E14.5', 'ICD-E14.7'	2
Hemiplegia or paraplegia	ICD-G04.1', 'ICD-G11.4', 'ICD-G80.1', 'ICD-G80.2', 'ICD-G81.x', 'ICD-G82.x', 'ICD-G83.0-ICD-G83.4', 'ICD-G83.9'	2
Renal disease	ICD-I12.0', 'ICD-I113.1', 'ICD-N03.2-ICD-N03.7', 'ICD-N05.2-ICD-N05.7', 'ICD-N18.x', 'ICD-N19.x', 'ICD-N25.0', 'ICD-Z49.0-ICD-Z49.2', 'ICD-Z94.0', 'ICD-Z99.2'	2
Cancer	ICD-C00.x-ICD-C26.x', 'ICD-C30.x-ICD-C34.x', 'ICD-C37.x-ICD-C41.x', 'ICD-C43.x', 'ICD-C45.x-ICD-C58.x', 'ICD-C60.x-ICD-C76.x', 'ICD-C81.x-ICD-C85.x', 'ICD-C88.x', 'ICD-C90.x-ICD-C97.x'	2
Severe liver disease	ICD-I85.0', 'ICD-I185.9', 'ICD-I186.4', 'ICD-I198.2', 'ICD-K70.4', 'ICD-K71.1', 'ICD-K72.1', 'ICD-K72.9', 'ICD-K76.5', 'ICD-K76.6', 'ICD-K76.7'	3

Metastatic cancer	ICD-C77.x-ICD-C80.x'	3
HIV	ICD-B20.x-ICD-B22.x, ICD-B24.x'	6

Supplemental Table 3 – Modified Revised Cardiac Risk Index

RCRI subsystem	ICD-10 / OPS Codes
Ischemic heart disease	ICD-10: I20-I25
History of cerebrovascular disease	ICD-10: I60-I69
History of congestive heart failure	ICD-10: I11, I42.0, I42.1, I50
Insulin therapy	NA (subsystem omitted)
Kidney disease	ICD-10: N03, N04, N17-N19, R34, I12, I13, Z99.2
High risk surgery	See Table OPS Mortality Risk

Supplemental Table 4 – Model hyperparameters

Model	Hyperparameter	Value Range
Imputation - IterativeImputer	sample_posterior	[True, False]
	n_nearest_features	[10, 20, None]
	max_iter	[3, 5, 10, 20]
Imputation - KNNImputer	n_neighbors	[5, 10, 20, 30, 50]
EBM	outer_bags	randint(1, 50)
	inner_bags	randint(1, 50)
	max_bins	randint(2, 500)
	early_stopping_rounds	randint(10, 100)
	early_stopping_tolerance	[1e-6, 1e-5, 1e-4, 1e-3, 1e-2, 1e-1]
	max_rounds	[2500, 3000, 3500, 4000, 4500, 5000, 5500, 6000, 6500, 7000, 7500]
	interactions	[0.1, 0.5, 0.9]
	max_leaves	[2, 3, 4, 5]
Logistic Regression	C	[100, 10, 1.0, 0.1, 0.01, 0.001]
	_max_iter	randint(200, 1000)

	penalty	solver ‘liblinear’: ['l1', ‘l2’]<br><br>solver in ['lbfgs', ‘newton-cg’, ‘newton-cholesky’, ‘sag’]: ['l2', None]<br><br>solver 'saga': ['l1', 'l2', 'elasticnet', None]
RandomForest	n_estimators	[10, 25, 50, 100, 500, 1000]
	max_depth	[3, 6, 9, 15, None]
	criterion	["gini", "entropy", "log_loss"]
	min_samples_split	[2, 5, 10, 15]
	max_features	["sqrt", "log2", None]
XGBoost	n_estimators	randint(10, 1000)
	max_depth	randint(1, 12)
	learning_rate	uniform(loc=0.01, scale=0.5)
	colsample_bytree	uniform(loc=0.5, scale=0.5)
	subsample	uniform(loc=0.6, scale=0.4)
	reg_lambda	[1, 2, 3, 5, 10]
	gamma	[0, 1, 2, 3, 5, 10]

Supplemental Table 5 – Model performance

Model	Random seed	AUROC (95%) CI	Balanced Accuracy	F1 Score	Precision	Recall	Specificity	Brier Score	Average Precision
Logistic regression	1234	0.758 (0.731-0.784)	0.684	0.465	0.361	0.651	0.716	0.141	0.304
Explainable boosting machine	1234	0.793 (0.769-0.816)	0.709	0.5	0.404	0.657	0.761	0.132	0.333
XGBoost	1234	0.740 (0.711-0.769)	0.654	0.44	0.398	0.49	0.818	0.152	0.296
Random forest classifier	1234	0.753 (0.727-0.779)	0.662	0.451	0.41	0.501	0.822	0.141	0.304
Logistic regression	34545	0.788 (0.765-0.811)	0.704	0.494	0.393	0.664	0.744	0.135	0.329
Explainable boosting machine	34545	0.808 (0.786-0.831)	0.719	0.517	0.422	0.667	0.772	0.13	0.348
XGBoost	34545	0.780 (0.755-0.804)	0.696	0.5	0.446	0.569	0.823	0.144	0.34
Random forest classifier	34545	0.772 (0.746-0.796)	0.675	0.472	0.426	0.529	0.822	0.137	0.32
Logistic regression	456567	0.755 (0.729-0.781)	0.671	0.45	0.375	0.563	0.779	0.138	0.295
Explainable boosting machine	456567	0.775 (0.751-0.799)	0.693	0.472	0.376	0.634	0.753	0.134	0.308
XGBoost	456567	0.727 (0.701-0.752)	0.657	0.44	0.409	0.476	0.838	0.153	0.295
Random forest classifier	456567	0.755 (0.731-0.779)	0.645	0.422	0.392	0.456	0.834	0.136	0.283
Logistic regression	34547	0.772 (0.746-	0.701	0.505	0.415	0.645	0.756	0.141	0.343

		0.798)							
Explainable boosting machine	34547	0.797 (0.773-0.820)	0.702	0.519	0.47	0.578	0.826	0.136	0.361
XGBoost	34547	0.783 (0.758-0.807)	0.707	0.52	0.452	0.612	0.801	0.137	0.359
Random forest classifier	34547	0.775 (0.751-0.800)	0.693	0.502	0.437	0.59	0.797	0.139	0.344
Logistic regression	4504569	0.742 (0.715-0.769)	0.668	0.446	0.371	0.56	0.777	0.143	0.291
Explainable boosting machine	4504569	0.766 (0.741-0.793)	0.664	0.45	0.414	0.493	0.836	0.137	0.3
XGBoost	4504569	0.742 (0.714-0.769)	0.653	0.433	0.398	0.476	0.83	0.142	0.289
Random forest classifier	4504569	0.762 (0.737-0.788)	0.654	0.441	0.445	0.437	0.872	0.134	0.302

Supplemental Table 6 – Model performance by gender

	Male	Female	p value
**Balanced Accuracy**	0.677 ± 0.025	0.684 ± 0.028	0.294
**AUROC**	0.756 ± 0.027	0.779 ± 0.02	< 0.05
**F1 Score**	0.473 ± 0.04	0.47 ± 0.032	0.705
**Precision**	0.403 ± 0.039	0.422 ± 0.024	< 0.05
**Recall**	0.58 ± 0.074	0.541 ± 0.087	< 0.05
**Specificity**	0.774 ± 0.043	0.827 ± 0.038	< 0.05
**Average Precision**	0.321 ± 0.034	0.314 ± 0.02	0.29
